# Prevalence and fecal egg load of gastrointestinal parasites of Angora goats in four agro-ecological zones in Lesotho

**DOI:** 10.14202/vetworld.2021.339-346

**Published:** 2021-02-05

**Authors:** Leballo G. Matsepe, Setsumi Molapo, Moeketsi Phalatsi, Mamajone Phororo

**Affiliations:** 1 Department of Animal Science, National University of Lesotho, P.O. Roma 180, Maseru, Lesotho; 2 Department of Biology, National University of Lesotho, P.O. Roma 180, Maseru, Lesotho

**Keywords:** fecal egg counts, gastrointestinal parasites, goats, *Haemonchus contortus*, prevalence

## Abstract

**Background and Aim:**

Goats are reared for their meat, mohair and other socio-cultural needs in Lesotho. Helminth infections are some of the major setbacks in the goat production industry due to their negative impact on animals’ health, resulting in significant losses on meat and mohair production and death. A cross-sectional study was conducted to determine the prevalence, fecal egg infestation, and morphological identification of gastrointestinal parasites in goats.

**Materials and Methods:**

Fecal samples were collected from 765 goats and subjected to McMaster egg counting techniques using the flotation method. Statistical analyses were performed using the Statistical Package for the Social Sciences (SPSS v.26.0).

**Results:**

The overall prevalence of gastrointestinal parasites was 94.7%, and the identified gastrointestinal parasites were nematodes (64.7%), coccidia (25.8%), and cestodes (4.2%). *Haemonchus contortus* was identified as the prevalent gastrointestinal nematode species found in goats. The prevalence and fecal egg count of gastrointestinal parasites were significantly higher (p<0.05) in goats located in the highlands and Senqu River Valley, while goats in the lowlands demonstrated a significantly (p<0.05) higher prevalence of *H. contortus*. Immature goats and kids were more significantly (p<0.05) prone to gastrointestinal parasites.

**Conclusion:**

The nematodes and coccidia infestations were prevalent in goats located in the highlands and foothills, respectively, whereas nematode and coccidia fecal egg loads were higher in goats located in the foothills and Senqu River Valley, respectively.

## Introduction

Angora goat rearing is a traditional Basotho farmers’ activity and plays a key role in their nutrition, social, and economic needs. Angora goat production serves as a cushion in the event of crop failure due to climatic vagaries, especially in arid and semi-arid environments [1,2]. Angora goats provide an essential income source to farmers by selling their mohair and meat or the animals themselves [3-5]. However, goat production faces multiple challenges, such as harsh climatic conditions, poor management and scarcity of fodder, and infectious and noninfectious diseases. In addition, Bath *et al*. [6] stated that despite Angora goats being renowned for their mohair production, they are susceptible to several diseases. Among the diseases, parasites are more problematic in the developing world, mainly where nutrition and sanitation standards are low. Moreover, Hoste *et al*. [7] indicated that parasitic nematodes of the gastrointestinal tract (GIT) are the main constraints to goat production worldwide and a significant health issue in goat rearing areas with poor sanitation and management. Gastrointestinal parasites remain a primary constraint to ruminant production since they can reduce in skeletal growth, live-weight gain, and milk yield [8].

Furthermore, Ademola *et al*. [9] reported gastrointestinal nematode (GIN) parasitism as the most serious constraint affecting ruminant production. Dappawar *et al*. [1] also stated the browsing habit of goats and their nomadic nature of husbandry as the most factors exposing them to parasitic infestation. Therefore, the GIT of animals harbors a wide variety of parasites, such as helminths, coccidia, and more, which cause clinical and subclinical parasitism. Zanzani *et al*. [10] also reported that GINs are important helminth groups that cause direct damage to livestock.

A cross-sectional study was conducted to determine the prevalence, fecal egg infestation, and morphological identification of gastrointestinal parasites in goats.

## Materials and Methods

### Ethical approval

The ethical approval was granted by the Department of Animal Science of the National University of Lesotho based on the recommended principles for the use of animals in conducting research. The animals were used with the consent of the farmers.

### Study area and period

**Figure-1:**
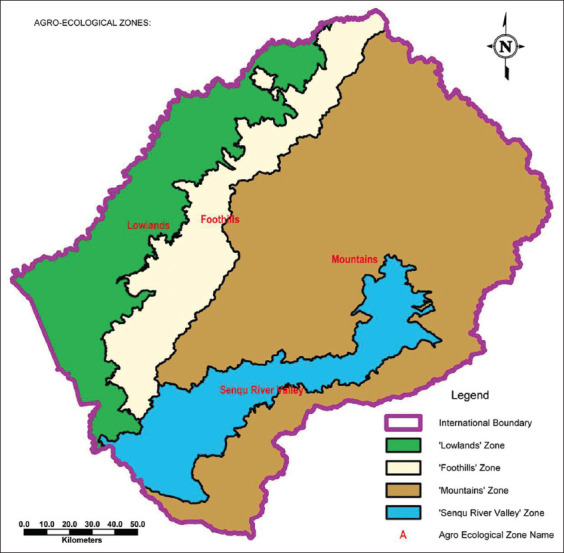
Agro-ecological Zones [Source: Lesotho Ministry of Public Works and Transport].

The fecal sample collection was conducted from December 2018 to October 2019 in four agro-ecological zones of Lesotho which are Senqu River Valley, Highlands (Mountains), Foothills and lowlands represented by Qacha’s Nek, Thaba-Tseka, Leribe and Mafeteng districts, respectively (Figure-1). Senqu River Valley is a narrow strip of land lying between an altitude of 1400 m and 1800 m and situated at 30º02’46.03” S and 28º21’40.79” E. The Highlands are the largest ecological region occurring at an elevation of 2000 m to 3400 m with coordinates of 29º31’59.85” S and 28º16’7.04” E. The Foothills are a strip of land lying between 1800 m and 2000 m above sea level with the coordinates of 29º00’3.79” S and 28º12’36.53” E whereas the lowlands are a narrow belt of land where elevation ranges from 1400 m and 1800 m with coordinates of 29º36’2.32” S and 27º17’32.71” E [11,12].

### Experimental animals

A total of 765 experimental animals kept under a semi-intensive management system were randomly selected. Animals <1 year (0-12 months) were considered kids, while those >1 year up to 2 years were immature (12-24 months), and those above 2 years were considered adults (>24 months).

### Study design and sampling method

The simple random sampling technique was employed in the four agro-ecological zones of Lesotho. Four villages in each agro-ecological zone were selected with the assistance of shearing shed farmer associations. Three goat farmers were randomly selected in each agro-ecological zone, whereby each farmer provided 15 animals as experimental units composed of five kids, five immature, and five adult goats.

### Fecal sample collection

Fecal samples were collected directly from each animal’s rectum using sterile disposable plastic gloves and were placed in the labeled screw-capped plastic bottles and then kept in a cooler box with ice packs. The samples were then transported to the National University of Lesotho and refrigerated at 4°C, and the laboratory analyses were performed within 48 h.

### Fecal sample examination

Two grams of crushed fecal samples were mixed with 58 ml of sodium chloride (flotation solution) and stirred. A homogenous solution was strained into the beaker, and few drops of amyl alcohol (3-5 drops) were added to treat bubbles in the solution. A McMaster quantitative technique was used to determine the number of eggs present per gram of feces, and each number obtained was multiplied by a factor of 100 to give an approximate number of eggs/gram of feces. A sub-sample was drawn from each sample using disposable pipettes and filled both chambers of the McMaster slides, which were observed under a microscope at 100× following standard procedures outlined by several researchers [13-16].

### Preparation of fecal cultures and harvesting of infective larvae

Copro-cultures comprising pooled samples from each agro-ecological zone were prepared using goat fecal pellets. The pellets were thoroughly crumbled before being mixed with sufficient vermiculite chips to yield a crumbly mixture, which was lightly compacted and moistened sufficiently. The crumbly mixture was ensured not to be water-logged and then incubated in jars in the dark (laboratory cupboards) at room temperature for 7 days. The inside of jars was sprayed lightly with water before being placed in bright light that stimulates the L_3_ to migrate up the inner walls of culture jars and then harvested repeatedly by holding the jars at a slant position with mouth pointing downwards and spraying the inner walls with water to allow the larvae to drain into a suitable container (beaker) as recommended by van Wyk and Mayhew [17].

### Larvae preservation

The larvae were preserved unchanged by adding formalin to the larval suspension to a final concentration of about 1-2% and then heated to 57°C in a water bath for about 1 min to kill larvae and straighten or uncurl it [17,18].

### Larvae preparation for identification

Larvae were prepared for examination by adding a drop of diluted Lugol’s iodine solution to a drop of larval suspension on a glass microscope slide and then mounted a coverslip as outlined by van Wyk *et al*. [18].

### Larvae identification

Morphological identification of L_3_ of most parasitic nematodes was based principally on examining the caudal (tail) and cranial (head) extremities. Conventional characteristics for identification (total length, sheath tail extension [STE] length, and filament length) of infective larvae GIN species were microscopically examined at 10× with ocular number ten and measured using a calibrated stage micrometer that was imputed in an ocular lens [17].

### Statistical analysis

The data collected were manually inputted in Microsoft Excel spreadsheet and transferred into SPSS v.26.0 for analyses. General linear model was employed to determine the effect of agro-ecological zone and age on the prevalence and fecal egg load of gastrointestinal parasites in goats. Generalized estimating equation (GEE) was used to analyze the FEC, where negative binomial regression was involved in the analysis. Odds ratio was used to measure the association between exposure and outcome. Descriptive statistics were implemented to determine the prevalence of identified species between the animals’ agro-ecological zones and age groups. Pearson Chi-square test was also adopted to assess the degree of association between each risk factor and GIN. In the analyses, the confidence level was held at 95%.

## Results

The goats in the highlands and foothills were more susceptible to nematode infestation than those in the lowlands and Senqu River Valley (Table-1). The prevalence of nematodes in goats in the lowlands was significantly (p<0.05) lower, and the chances of nematode infestation from the lowlands to the foothills, highlands, and Senqu River Valley increased significantly (p<0.05).

**Table-1:** Prevalence of gastrointestinal parasites by agro-ecological zones.

Agro–ecological zones	No. examined	Prevalence (%)	SE	Exp. (B)	Sig. Level
Nematodes					
Senqu river valley	1 251	64.7^a^	0.014	0.771	0.000
Highlands	1 533	70.0^b^	0.012	0.607	0.000
Foothills	1 724	67.1^ab^	0.011	0.696	0.000
Lowlands	1 986	58.6^c^	0.011	1	
Coccidia					
Senqu river valley	1 251	26.3^a^	0.012	0.650	0.000
Highlands	1 533	25.4^a^	0.011	0.680	0.000
Foothills	1 724	33.6^b^	0.011	0.458	0.000
Lowlands	1 986	18.8^c^	0.009	1	
Cestodes					
Senqu river valley	1 251	5.3^a^	0.006	0.588	0.003
Highlands	1 533	4.2^ab^	0.005	0.752	0.115
Foothills	1 724	4.6^a^	0.005	0.682	0.028
Lowlands	1 986	3.2^b^	0.004	1	

abc Means in the same column with different superscripts differ significantly (p<0.05). Exp. (B)=Exponential Beta, No.=Number, Sig.=Significant level

As illustrated in Table-1, the prevalence of coccidia infestation showed that the infestation was significantly (p<0.05) higher in the foothills. The results also demonstrated that the likelihood of increased coccidia infestation from the lowlands to the foothills, highlands, and Senqu River Valley was significant (p<0.05).

Goats in the lowlands were significantly (p<0.05) different from those in the highlands in the prevalence of cestodes. As depicted in Table-1, these results also displayed that the odds of having cestodes infestation from lowlands to foothills and Senqu River Valley increased significantly (p<0.05). However, the likelihood of having a cestode infestation from the lowlands to highlands increased insignificantly (p>0.05).

The prevalence of nematodes was high (p<0.05) in immature goats, followed by adults, and the lowest prevalence was observed in kids (Table-2). The odds of having a nematode infestation from adult goats to immature goats increased insignificantly (p>0.05), while from adults to kids, odds decreased significantly (p<0.05).

**Table-2:** Prevalence of gastrointestinal parasites by age.

Age	Samples examined	Prevalence (%)	SE	Exp. (B)	Significance level
Nematodes					
Kids	2 182	58.2^a^	0.01	1.47	0.00
Immature	2 101	69.1^b^	0.01	0.91	0.16
Adults	2 211	67.1^b^	0.01	1	
Coccidia					
Kids	2 182	33.0^a^	0.01	0.51	0.00
Immature	2 101	24.2^b^	0.01	0.79	0.01
Adults	2 211	20.1^c^	0.01	1	
Cestodes					
Kids	2 182	6.6^a^	0.005	0.332	0.00
Immature	2 101	3.6^b^	0.004	0.629	0.01
Adults	2 211	2.3^c^	0.003	1	

abc Means in the same column with different superscripts differ significantly (p<0.05). Exp. (B)=Exponential beta

In the case of coccidian (Table-2), the infestation was found significantly (p<0.05) lower in adult goats than in immature and young goats. The likelihood of coccidia infestation increased significantly (p<0.05) from adult goats to immature goats. Furthermore, from adults to kids, the probability of coccidia infestation also increased significantly (p<0.05).

In terms of cestodes, a high prevalence was observed in kids, followed by immature goats, and was lowest in adult goats. There was also a significant difference (p<0.05) discovered between the different age groups. The likelihood of goats exhibiting cestodes infestation from adults to immature and kids increased significantly (p<0.05). The low prevalence of cestode infestation observed in adults might be due to body resistance as they might have developed immunity due to repeated natural infestations.

**Figure-2:**
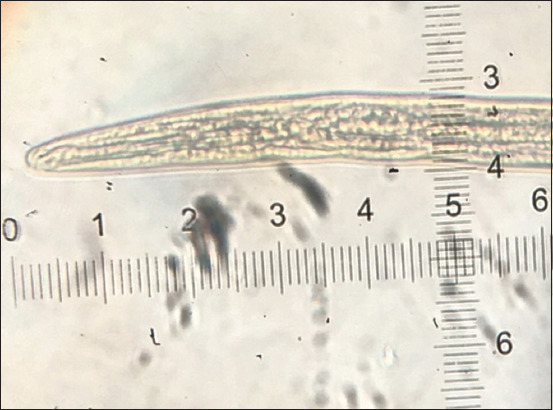
Head shape.

**Figure-3:**
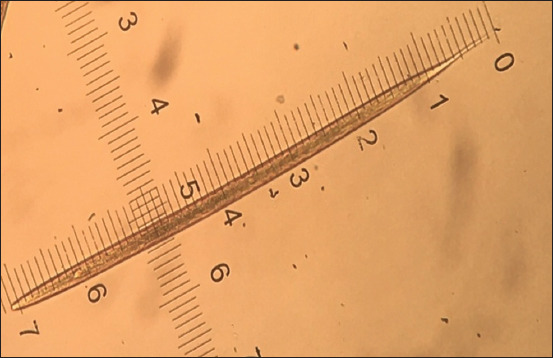
Typical *Haemonchus* spp.

*Haemonchus contortus* was the only species of GI nematodes that were discovered in this study. Of 172 samples examined, 130 were positive for *H. contortus*, and the overall prevalence in goats in this study was 75.6%. The cranial extremity of the infective larvae was observed to have a bullet-shaped head (Figure-2). According to van Wyk and Mayhew [17], the length of STE is an essential criterion for identification; therefore, the caudal extremity, which comprises a sheath tail filament, ranged following the report of van Wyk *et al*. [18], which was 2.2-2.5 μm and 10-15% for STE and filament, respectively. van Wyk *et al*. [18] van Wyk *et al*. [17] further emphasized that *H. contortus* had an STE of >2.0 μm, with 10-15% filament, head bullet-shape, and temper fast to a point (Figure-3).

The higher (p<0.05) prevalence of *H. contortus* observed in the lowlands (Table-3) compared with other agro-ecological zones suggests that the environmental conditions were favorable for the development and survivability of infective larvae (*H. contortus*) and lack of shrubs for goats to browse; hence, forcing the goats to graze closer to the ground, thereby, contacting the infective larvae. A higher (p<0.05) *H. contortus* infestation was observed in kids than in adult and immature goats.

**Table-3:** Prevalence of *Haemonchus contortus* in goats by different agro–ecological zones and age.

Category	No. examined	Positive cases	Prevalence (%)	χ^2^– value	p-value
Agro-ecological zones					
Senqu River Valley	41	34	82.9	31.745	0.000
Highlands	40	26	65.0		
Foothills	40	20	50.0		
Lowlands	51	50	98.0		
Age of goats					
Kids	64	55	85.9	5.939	0.051
Immature	58	40	69.0		
Adults	50	35	70.0		

χ^2^ – Pearson Chi-square value, P – Significance level

A higher (p<0.05) intensity of nematode FEC mean was observed in goats located in the foothills compared with other agro-ecological zones (Table-4). The likelihood of coccidia infestation increased significantly (p<0.05) from the lowlands to the foothills and from the lowlands to Senqu River Valley but decreased significantly (p<0.05) from the lowlands to the highlands. Our findings also displayed that the cestodes egg infestation from the lowlands to the foothills increased significantly (p<0.05) while from the lowlands to Senqu River Valley significantly (p<0.05) decreased.

**Table-4:** Means of gastrointestinal FEC in different agro–ecological zones.

Agro–ecology	EMM	SE	Exp (B)	Exp (B) in %
Nematodes				
Senqu river valley	440.13^ab^	36.18	1.419	41.9
Highlands	453.36^a^	21.99	1.461	46.1
Foothills	542.52^a^	85.92	1.749	74.9
Lowlands	310.22^b^	22.74	1	1
Coccidia				
Senqu river valley	927.18^a^	463.47	5.756	475.6
Highlands	133.68^b^	17.83	0.830	17.0
Foothills	329.41^c^	57.35	2.045	104.5
Lowlands	161.08^d^	33.52	1	1
Cestodes				
Senqu river valley	33.81^a^	10.27	0.813	18.7
Highlands	120.03^b^	57.95	2.886	188.6
Foothills	56.32^a^	27.81	1.354	35.4
Lowlands	41.59^a^	13.70	1	1

abc Means in the same column with different superscripts differ significantly (p<0.05). EMM=Estimated marginal means, SE=Standard error, Exp.(B)=Exponential Beta

The nematode egg load was higher in immature goats, followed by adult goats, and lowest in kids (Table-5). The adopted GEE revealed that nematode FEC increased significantly (p<0.05) from adults to immature goats, whereas from adults to kids, the likelihood of infestation decreased significantly (p<0.05).

**Table-5:** Means of gastrointestinal FEC in different age groups of goats.

Age	EMM	SE	Exp. (B)	Exp. (B) in %
Nematodes				
Kids	323.19^a^	16.01	0.712	28.8
Immature	517.61^b^	67.82	1.140	14.0
Adults	454.23^c^	34.00	1	
Coccidia				
Kids	723.69^a^	262.41	8.944	794.4
Immature	235.36^b^	70.45	2.909	190.9
Adults	80.91^b^	16.41	1	
Cestodes				
Kids	165.95^a^	47.28	34.944	3 394.4
Immature	15.90^b^	9.71	3.347	234.7
Adults	4.75^c^	1.38	1	

abc Means in the same column with different superscripts differ significantly (p<0.05). EMM=Estimated marginal means, SE=Standard error, Exp.(B)=Exponential bet

This study showed that adult goats were significantly lower (p<0.05) than other age groups in terms of coccidia fecal egg load. The study further demonstrated that coccidia egg count was significantly (p<0.05) higher in kids, followed by immature goats, and lowest in adult goats.

The kids had significantly (p<0.05) higher cestode egg load than immature and adult goats. The likelihood of infestation increased significantly (p<0.05) from adult to immature goats, and from adults to kids, the chances of infestation increased significantly (p<0.05).

## Discussion

This study’s results agree with the findings obtained by Koinari *et al*. [19], who reported a high prevalence of *Strongyloides* (nematodes) in goats in the highlands. This high prevalence of nematodes, coccidian, and cestodes observed in goats in the highlands, foothills, and Senqu River Valley, respectively, suggests the existence of favorable environmental conditions for survival and development of parasitic larvae to the infective stage. Nabi *et al*. [4] illustrated that the prevalence of GIT parasites varies in diverse geographical conditions and is influenced by climate, management, vegetation, and livestock density. The low GIP prevalence in goats reared in the lowlands agrees with the observation of Mpofu *et al*. [2], who illustrated that the low prevalence of nematodes, coccidia, and cestodes in arid zones (lowlands) might be attributed to the fact that these areas are extremely hot and receive scarce, erratic rainfall, which is unfavorable for GIP development, survival, and transmission.

However, in a study by Moiloa *et al*. [3], nematode infestation was higher in the lowlands of Maseru district and the foothills of Quthing district, while coccidia infestation was highest in the mountains of both Maseru and Quthing districts. Berhe and Aragaw [20] further emphasized that the higher parasitic infestation suggests that goats, which are typically browsers, may have been forced to graze, perhaps due to lack of adequate browsing material in the study area. Emiru *et al*. [8] emphasized that the high prevalence could be ascribed to overstocking, poor nutrition (starvation), poor management practice of the animals (lack of sanitation), and frequent exposure to the communal grazing lands contaminated, which agrees with the findings of this study since pastures are communally grazed. According to a study conducted by Dagnachew *et al*. [21], there was a statistically significant (p<0.05) difference in the species prevalence of helminthiasis, with the highest being in Chillga (lowland) for strongyle infestation (55.45%) and the lowest was in Dabat (highland) (22.52%). In addition, Khodakaram-Tafti and Hashemnia [22] emphasized that the variation in the prevalence and distribution of coccidiosis may be attributed to, among other factors, agroecology. Moreover, Rahman *et al*. [23] also outlined that the lower prevalence of *Moniezia* spp. (cestodes) might be due to less dissemination of eggs in the feces from the gravid segment.

The results of this study agree with those reported by Sunandhadevi *et al*. [16], who showed that the prevalence of GI nematode was more in immature (84.44%) than in adult (64.22%) and young goats (64.10%). Islam *et al*. [24] Mpofu *et al*. [2] also added that infestation with GIPs was more prevalent in immature goats than in adult goats and kids. This high prevalence of GI nematodes obtained in immature goats can be attributed to the fact that goats were kept in a contaminated environment, overstocked, and had low immunity. However, despite the above inference drawn, Dagnachew *et al.*, [21] Nabi *et al.*, [4] noted the significant difference between young and adult goats, in which young goats showed higher nematode infestation than adults. Mpofu *et al*. [2] also argued that young goats showed a higher incidence of nematode infestation than adult goats. Nabi *et al*. [4] emphasized that with an increase in age, the prevalence of GIT nematodes decreased due to the development of immunity, and it has been reported that small ruminants develop partial immunity against GIT nematodes. Furthermore, Lone *et al*. [25] stated that the most infested age group was kids (97.77%). Alternatively, Ardo and Bitrus [26] argued that adult animals were more infested than the young goats, whereas Yusof and Isa [27] viewed that the highest prevalence of GINs infestation was observed in immature (87.8%), followed by the adults (86.4%) and kids (53.1%).

According to the present findings, Gupta *et al*. [28] reported that the overall prevalence of gastrointestinal parasites was 98.05% of which the highest percentage was *Eimeria* spp. (coccidia) (86.34%). This may be attributed to the fact that kids are underdeveloped and have lower immunity resistance toward coccidia infestation than immature and adult goats. Similarly, Jittapalapong *et al*. [29] also reported that a higher GI parasitic prevalence was observed among goats aged <1 year (94.9%) than goats aged 1-2 years (75.4%) or >2 years old (51.4%). Zvinorova *et al*. [30] added that *Eimeria* (coccidia) infestation was the most prevalent parasitic infestation, followed by *Strogyles* (nematodes). Nevertheless, in a study conducted by Yusof and Isa [27], adult goats’ well-developed resistance appeared to be relative rather than absolute since adults continued to harbor *Eimeria* spp. oocysts in feces, which contributed as a source of infestation for younger goats. Moreover, Mpofu *et al*. [2] added that adult animals may acquire immunity to the parasite through frequent challenges and may expel the ingested parasite before an infestation is established, thereby contaminating the environment.

These results also agree with the study conducted by Yusof and Isa [27], who noted that the prevalence of tapeworm (cestodes) was higher in young and juvenile age groups than adult animals. Furthermore, Raza [31] highlighted that the host animal’s age was an essential factor influencing the prevalence of GI parasites.

The results of this study agree with the findings of a previous study in Papua New Guinea [19], who discovered that the mean EPG counts for goats infested with *Strongyloides* (nematodes) in the highlands (Tambul) were significantly higher than those in the lowlands (Labu). Koinari *et al*. [19] also added that the mean EPG for *Eimeria* was higher in the highlands than in the lowlands. However, Regassa *et al*. [32] argued that higher values were recorded for the lowland areas, followed by mid-altitude areas with the lowest values in the highland areas.

Sharma *et al*. [33] also observed the highest (1 690.1 EPG) number of oocysts in the immature age group, whereas a minimum (22.73 EPG) oocysts load was found in kids that were still suckling. This high nematode infestation of the immature age group than in kids and adult goats might be influenced by the weaker immunological response of immature goats, thereby becoming susceptible to GINs.

Following the results of this study, Kahan and Greiner [34] also added that the EPG of kids was significantly higher than in adult goats (p<0.01). These findings illustrated that the number of oocysts shed decreased as the animal matured. Kahan and Greiner [34] further added that this decline is most likely due to the development of an immunological resistance related to host’s age and exposure to the parasites. In contrast with the current study, Kheirandish *et al*. [35] reported that adult goats had higher oocyst counts than kids and immature goats.

The findings in this study agree with the report of Yusof and Isa [27], who outlined that cestode infestation was prevalent in kids, and because of the low pathogenicity in adults, tapeworm (cestodes) infestation was considered minor.

## Conclusion

It can be concluded that nematode and coccidia infestation was higher in the highlands and foothills, while nematodes and coccidia fecal egg loads were higher in foothills and Senqu River Valley. *H. contortus* was identified as the prevalent GIN species found in goats. Nematodes and coccidia highly infested immature and kids.

## Authors’ Contributions

LGM conceived and designed the research under the guidance of SM. LGM, MoP, and MaP conducted the sample collection. MoP and LGM carried out the morphological identification analysis. SM, MaP, and LGM carried out the data analyses. LGM wrote the manuscript. SM reviewed the manuscript. All authors read and approved the final manuscript.
